# Increasing spring temperatures favor oak seed production in temperate areas

**DOI:** 10.1038/s41598-017-09172-7

**Published:** 2017-08-17

**Authors:** Thomas Caignard, Antoine Kremer, Cyril Firmat, Manuel Nicolas, Samuel Venner, Sylvain Delzon

**Affiliations:** 1 BIOGECO, INRA, Univ. Bordeaux, 33615 Pessac, France; 2 INRA, URP3F, RD150, Site du Chêne, BP 86006, 86600 Lusignan, France; 3 Office National des Forêts, Département recherche-développement-innovation, Boulevard de Constance, 77300 Fontainebleau, France; 4 Laboratoire de Biométrie et Biologie Evolutive UMR 5558-CNRS, Université de Lyon, Université Claude Bernard Lyon 1, Villeurbanne, F-69365 Lyon France

## Abstract

The changes in reproductive phenology (i.e. timing of flowering and fruiting) observed in recent decades demonstrate that tree reproduction has already been altered by climate change. However, understanding the impact of these changes in reproductive success and fitness remains a major challenge for ecologists. We describe here a previously unreported phenomenon: a significant increase in the reproductive effort (seed production) of temperate oaks with increasing spring temperature, observed over the last decade. In contrast, no relationship was found between seed production and precipitation. This sensitivity of seed production to temperature was confirmed by a “space-for-time” substitution based on elevation gradients. Our findings suggest that global warming may enhance oak reproductive effort in temperate ecosystems. Nevertheless, while fitness can be enhanced by higher levels of seed production, it also depends on the frequency and synchronization of mast seeding production, which may also be influenced by climate change.

## Introduction

Forests are important for biodiversity and as a terrestrial carbon sink^1^, and contrasting responses to climate change have been identified. For instance, growth and survival, two of the main components of tree fitness, have been found to be substantially altered by climate change^2, 3^. In cold and mild areas, such as boreal and temperate forests, global warming is extending tree growing seasons^4, 5^ and promoting wood production and tree growth^3^, whereas, in warmer and drier areas, negative impacts on tree growth^6^ and survival^7, 8^ have been observed. In addition to the reported impact on growth, and, to a lesser extent, forest dieback, we need to know how tree reproduction, one of the most important components of plant fitness, is being affected by climate change, and its likely response.

Reproduction is critical for the maintenance and demography of populations, and should therefore be assessed carefully when modeling population responses to climate change^9^. Seedling regeneration and survival are directly linked to variations in seed production^10, 11^ and the assessment of changes in regeneration from seeds in response to temperature has become a major challenge. There is, therefore, an urgent need to assess the impact of climate change on tree reproduction, to improve our understanding of the likely effects of this phenomenon on tree population dynamics.

An impact of climate change on the timing of reproduction has been reported for numerous organisms^12, 13^. Indeed, reproductive phenology is known to be sensitive to environmental cues, such as temperature^14, 15^, so climate change is likely to alter the intensity of seed production. However, the impact of climate change on reproductive effort is difficult to quantify, particularly in forest trees, which display the synchronized, intermittent production of large amounts of seeds. This phenomenon, commonly observed in oak species at the population scale, is called “masting” or “mast-seeding”^16, 17^. Most studies of tree seed production over long time series have focused on single sites or small numbers of sites in limited areas. The specific features of masting have, thus, made it difficult to assess the sensitivity of seed production to temperature. Moreover, as pointed out by Crone and Rapp^18^, the large numbers of isolated studies and of weather variables tested have highlighted contradictory correlations with seed production, even for related species. As a result, to avoid artifacts caused by masting, the monitoring of seed production should be replicated in space and time, in ecologically independent forests.

In this study, we analyzed extensive sets of tree reproduction data for two temperate European white oak species (the sessile oak (*Quercus petraea*) and the pedunculate oak (*Q*. *robur*)), to determine whether seed production had changed over the last two decades in response to global warming. Seed production was monitored in 28 forests of *Q*. *petraea* and *Q*. *robur* distributed throughout France over a period of 14 years. In parallel, a “space-for-time” substitution was used to quantify the temperature sensitivity of acorn production over elevation gradients. These analyses demonstrated significant temperature-induced trends in seed production over the last two decades, suggesting that climate change enhances oak reproductive effort in temperate ecosystems.

## Results and Discussion

### Temporal trend in seed production

**Figure 1:**
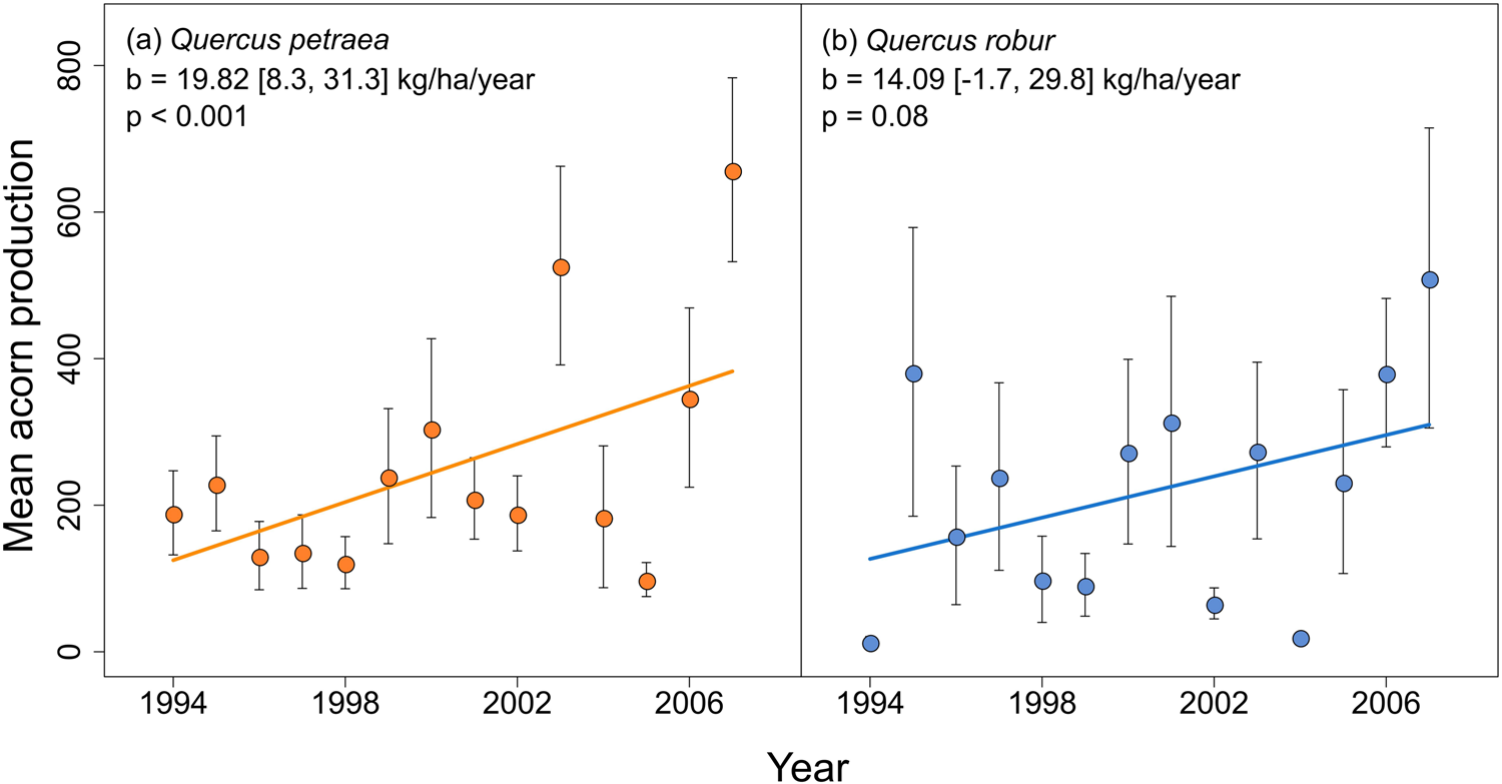
Temporal variation in seed production for *Quercus petraea* and *Quercus robur*. Temporal variation in seed production (kg/ha) of 19 and 9 populations of *Q*. *petraea* (**a**) and *Q*. *robur* (**b**) respectively, monitored over 14 years and distributed throughout France. Each dot corresponds to the mean seed production across all populations per year (kilograms per hectare per year averaged over all sites), the standard errors are indicated for each dot. The slope of the regression line and its 95% confidence interval, calculated from a linear mixed-effects model [2], are given for both species, with the coefficient of determination (*R* ^2^) between model [2] and mean production.

We examined temporal changes in the seed production of two oak species across France over recent decades (1994 to 2007) (Figure S1). We observed an increase over time in reproductive effort for both species (Fig. 1). On average, acorn production (M_acorn_) in *Q*. *petraea* populations increased by 19.8 [8.3, 31.3] kg/ha/year (Table 1) and by 14.1 [−1.7, 29.8] kg/ha/year for *Q*. *robur*. However, for *Q*. *robur* the regression slope was not significant (Table 1). In addition, the effects of age and diameter have been tested and are reported in Table S1. For both species, growth does not help to explain the variation in seed production (Table S1). However, aging had a significant negative effect on the fructification of *Q*. *petraea* (Table S2) while no effect was found for *Q*. *robur*. Such a negative effect suggests that older *Q*. *petraea* populations produce less acorns than younger ones. This strengthens the positive trend in seed production observed over time. Similar positive temporal trends have been reported in a few other studies. A limited number of reports for *Pinus engelmannii* ^19^ and in *Nothofagus solandri* ^20, 21^ have demonstrated temporal shifts. In these studies, the monitored populations were located at high elevations, at which reproduction appears to be more sensitive to environmental change^20, 21^. However, in most cases, no temporal trend in fruit production, for example, was observed^22, 23^ and such trends have rarely even been sought, due to the scarcity of adequate, long-term datasets. In our study, the many populations surveyed were found in temperate lowland forests located over a large area and at an elevation of between 55 and 330 m above sea level. The mean synchrony of seed production (Spearman correlation coefficient) among the populations was very low for both species (0.11 ± 0.016 for *Q*. *petraea* and 0.15 ± 0.052 for *Q*. *robur*), demonstrating the lack of synchrony between populations over this large scale (the differences in seed production dynamics between the populations monitored are shown in Figure S2). It is worth noticing that the degree of synchrony changes according to the distance between populations, explaining the low overall synchrony over the large distribution of the populations monitored in our study: the greater the distance between two populations is, the lower the synchrony (Figure S3). As the populations were independent, any common temporal change in reproductive effort can be seen as a robust overall pattern rather than a local trend in a marginal population. Many studies have explored the potential drivers of plant reproduction^18, 24, 25^, but only a few have investigated changes in reproductive effort in response to global warming, due to a general lack of statistical power^26, 27^. The large number of asynchronous populations monitored in our study provides a significant advance to assess changes in reproduction though time and according to temperature. In the context of climate change, the temporal trends observed here may reflect the effects of recent warming over the last few decades. Consistent with this view, we observed a significant increase in temperature over time at the sites studied (Figure S4), potentially sufficient to account for the positive temporal trend observed.

**Table 1:** Temperature sensitivity of reproductive efforts in oaks.

Species	Temporal		Spatial	
	M_Acorns_/Year	M_Acorns_/T_Ap-Ma_	M_Acorns_/Alt_100m_	M_Acorns_/T_Ap-Ma_
*Q*. *petraea*	**19.82 [8.3**, **31.3]**	**111.89 [63.1**, **146.0]**	**−83.89 [−149.5**, **−18.3]**	**334.2 [175.6**, **589.2]**
*Q*. *robur*	14.07 [−1.7, 29.8]	**72.66 [19.6**, **120.5]**	—	—

Slopes of the linear mixed-effect regression between acorn production in kilograms per hectare (M_Acorns_) and year (temporal gradient, (M_Acorns_/Year)), and for every 100 m increase in elevation (spatial gradient, M_Acorns_/Alt_100m_), and the mean temperature in April and May in °C (M_Acorns_/T_Ap-Ma_) in both studies. The 95% confidence intervals are indicated in square brackets. Reproduction in *Quercus petraea* was monitored in both studies, whereas *Quercus robur* was monitored in the temporal gradient study only. Significant correlations are indicated in bold.

### Reproductive effort in oak is increasing with increasing spring temperatures in temperate areas

**Figure 2:**
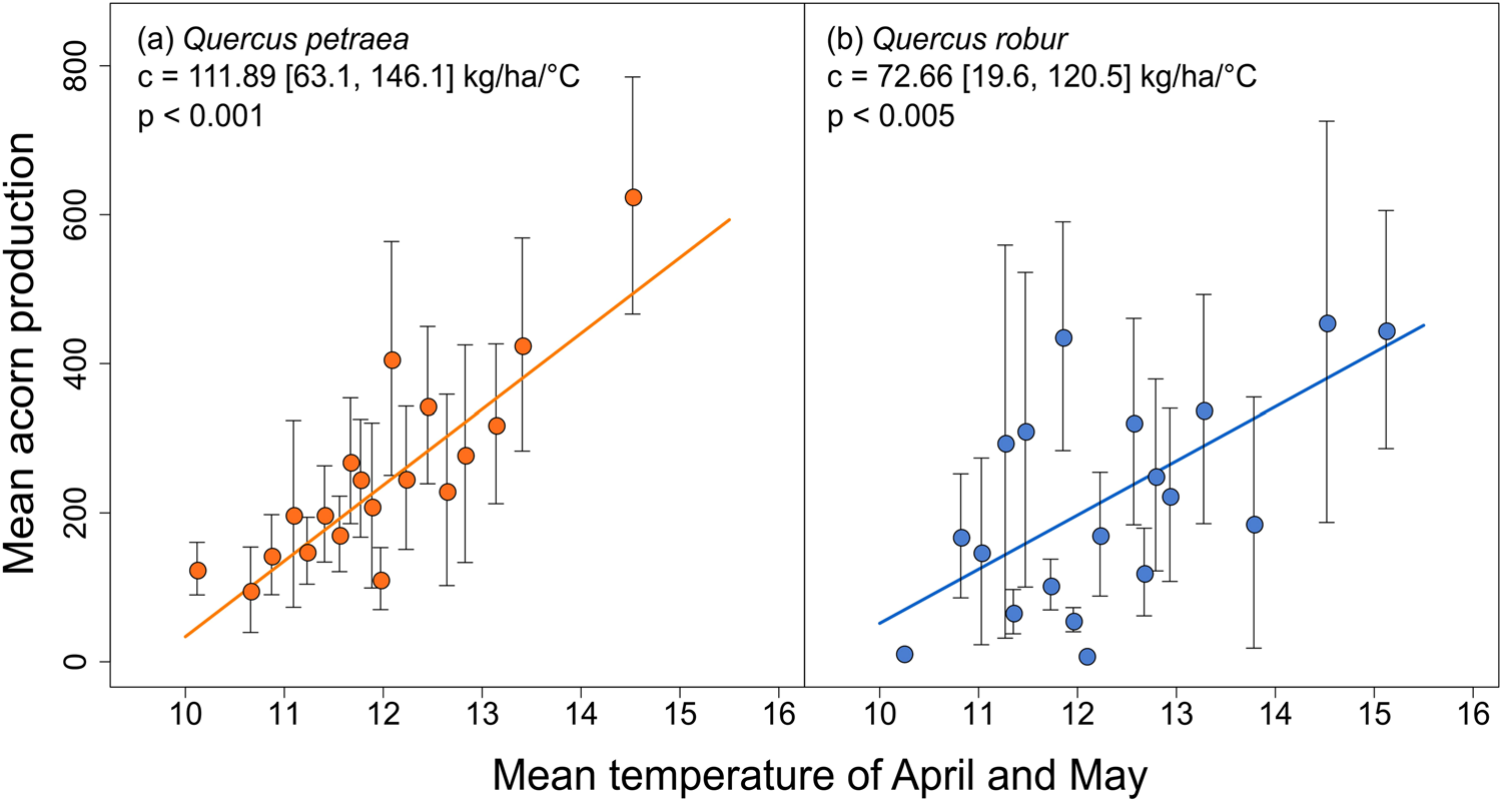
Responses of seed production to spring temperature for both *Quercus petraea* and *Quercus robur*. Changes in acorn production per population and per year (M_Acorns_) for *Q*. *petraea* (**a**) and *Q*. *robur* (**b**) according to mean spring temperature. For both species, acorn production data for all populations and all years were binned into 19 temperature classes of approximately the same size, 14 for *Q*. *petraea* and 6 or 7 for *Q*. *robur*. Mean acorn production per bin is correlated with the temperature class median, the standard errors are indicated for each dot. The slope of the regression line and its 95% confidence interval, calculated from a linear mixed-effects model [4], and the coefficient of determination (*R* ^2^) between the model [4] and the binned data are given for each species.

Temperature and rainfall are routinely recorded and are considered the most relevant climatic variables driving seed production^24^, but their effects seem to differ between tree species and ecosystems^23^. Tree reproductive effort has been studied mostly in Mediterranean oak species and monitored mostly in Southern Europe and California^28^. For most of the Southern European species, a warmer, drier summer season results in lower levels of seed production^29, 30^. Interestingly, the main driver appears to be water deficit rather than temperature *per se* ^31, 32^. By contrast, we found that, in both *Q*. *petraea* and *Q*. *robur*, seed production was positively correlated with spring temperature (Fig. 2a,b and Table 1), which is known to have a strong effect on flowering and pollination^14, 15^. Moreover, no correlation was found between seed production and annual or seasonal precipitations (Table S3). No study has ever reported positive temporal clines for acorn production, but positive correlations with spring temperature have been found in California for three Mediterranean oak species, *Q*. *lobota*, *Q*. *douglasii* and *Q*. *kelloggii* ^33^, and three temperate oak species, *Q*. *alba*, *Q*. *rubra* and *Q*. *velutina* ^17^. In our study, despite the broad distribution of the populations, the positive correlation with spring temperature observed could be explained mostly by temperature variability over time rather than temperature variability over space (Table S4). The trend towards an increase in seed production over time observed for both species was therefore directly correlated with the increase in spring temperature observed over the last decade (Figure S4). Climate change has had a negative impact on reproduction in Mediterranean oaks in Europe^32, 34^, but we show here that the increase in spring temperature has favored reproduction in temperate oaks. Such a difference could be explained by a water scarcity threshold that is annually reached in Mediterranean ecosystems, while precipitation is currently not limiting for reproduction in European temperate ecosystems.

We then examined seed production along elevation gradients in Southern France, to refine the temperature-seed production relationship. Our findings confirm the strong positive correlation between seed production and spring temperature in *Q*. *petraea* (Table 1). The gain in acorn production per one-degree rise along the elevation gradient (M_acorn_/T_Ap-Ma_ = 334.2 kg/ha/°C) was three times greater than that along the spatio–temporal gradient (M_acorn_/T_Ap-Ma_ = 111.89 kg/ha/°C). This difference may reflect differences in temperature values and gradients between the two designs. Indeed, the range of spring temperature variation was lower for the spatio-temporal gradient (6.2 °C) than for the elevation gradient (10.8 °C).

### What is the impact on tree fitness?

**Figure 3:**
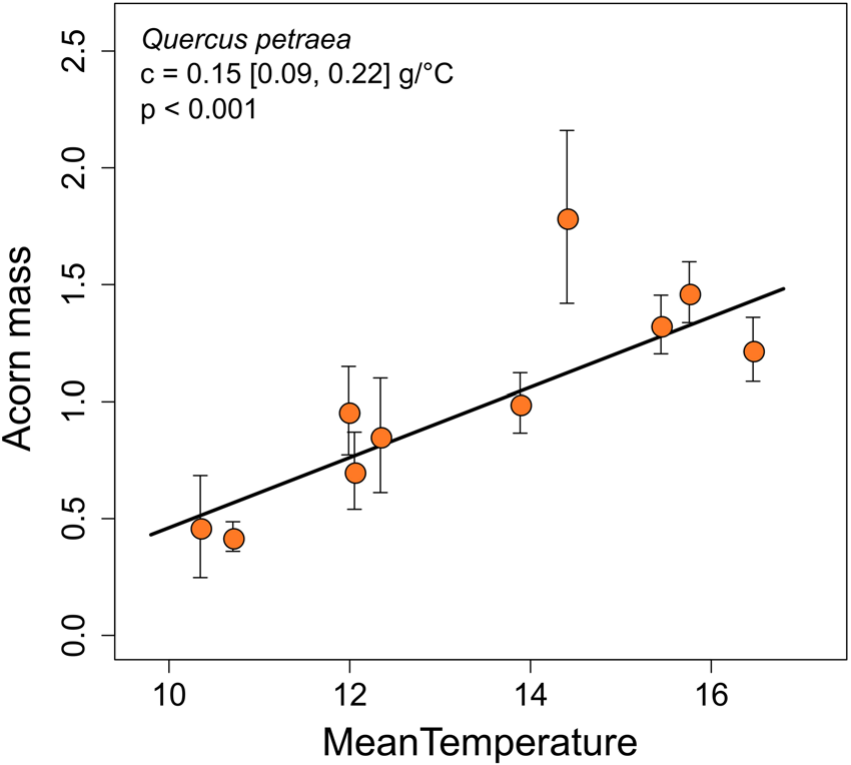
Temperature trends in seed mass for *Quercus petraea*. Trend in acorn mass (g) with mean temperature from April to November (°C) along the elevation gradient. Each dot corresponds to the mean acorn mass, across years and trees, per population along the elevation gradients. The slope of the regression line and its 95% confidence interval, calculated from a linear mixed-effects model, and the coefficient of determination (*R* ^2^) are indicated. April to November corresponds to reproductive cycle length in *Q*. *petraea*: from flowering to acorn release.

Our observations suggest that climate change may increase the fitness of temperate oaks. An increase in seed production is beneficial to the tree, as it increases seed dispersal^35, 36^, thereby increasing the number of potential offspring and, consequently, their establishment. In addition, acorn mass increases with increasing temperature, by about 0.15 g per degree [0.09, 0.22] (Fig. 3). This gain may increase the resistance of acorns to environmental stress (predation by insects, frost) and enhance germination^37, 38^. However, reproduction in many tree species, including oaks, is characterized by masting or mast-seeding, with synchronized large-scale seed production at the population scale (Figure S2). This process is considered to be an adaptive response to the selective pressure exerted by predators^16, 17^. Masting limits seed predation and promotes seed dispersal, thereby ensuring high rates of offspring survival and optimizing resource allocation to reproduction^24, 39^. Changes in masting associated with climate change may, therefore, have a negative impact on the fitness of tree populations.

Nowadays climate change effect on masting frequency is still uncertain and only few studies have investigated change in masting frequency and long-term shifts in allocation to seed production. For instance, Övergaard *et al*.^40^ observed, during 30 years of measurement in European beech forest (*Fagus sylvatica*), an increase in the frequency of mast events directly correlated with an increase in temperature. With increasing periodicity, the temporal variability characterizing mast-seeding and enabling the trees to control predator population size^24, 41^ might be greatly reduced^27^. Low inter-annual variability in seed crops may lead to an increase in predator population size, decreasing reduce offspring survival. This paradoxical consequence of climate warming for temperate tree reproduction highlights the need for improvements in our understanding of the proximal mechanisms underlying masting in trees, for prediction of the response of forest ecosystems to climate change.

This study focused on temperate forests dominated by deciduous oak species and cannot be extended to other forest types. However, we can compare the time and temperature trends of acorn production observed in oaks with the patterns reported for whole biomass growth in trees^42, 43^. There is a clear congruent increase in vegetative growth and reproductive growth (our results) in *Q*. *petraea* and *Q*. *robur* in recent decades in central Europe. Such trends can be seen as two facets of the overall consequence of the same causes relating to global changes in recent decades. An increase in temperature extends the period of vegetative growth^4, 5^ and enhances tree growth^44^. In addition to increasing temperature, increases in the carbon dioxide content of the atmosphere may also promote tree growth in some species, and increases in nitrogen (N) deposition have been shown to stimulate forest growth and carbon sequestration in Europe^45^. As reproduction in trees is also dependent on resource availability^46–49^, the combined effects of temperature, carbon dioxide, and nitrogen deposition may also contribute to the increase in seed production. Despite a likely competition for resources between these two processes, a concurrent increase in both is reliable as there seem to be largely independent of each other^50^. However, the congruent pattern of vegetative and reproductive growth may be negatively affected by extreme events and disturbances, such as firestorms or the spread of insects and diseases, which may also be triggered by global changes^51^.

Global warming has had a positive effect on temperate oak growth. However, the response of tree reproduction to environmental changes remains unclear, mostly due to our limited understanding of masting processes. Long-term studies of reproductive investment over large areas would be required to assess the global impact of climate change on trees.

## Material and Methods

### Study sites

We analyzed variations in seed production for two European oak species (Quercus petraea and Quercus robur) along a latitudinal and an elevation gradients. The latitudinal field survey is made of 28 permanent plots that are part of the French intensive forest monitoring network (RENECOFOR) (Figure S1). These plots are widely distributed across France, between latitudes of 43.2° and 50.2°N and longitudes of 0.04° and 3.7°E (Table S5). They correspond to oak tree populations dominated by *Q*. *petraea* for 19 of them and by *Q*. *robur* for the 9 other ones. All of these populations were already mature when they started to be monitored (mean age of 85.5 years ± 28.7 in 1994). Seed production was assessed for 14 years, from 1994 to 2007. Due to a budget cut, the monitoring program stopped after 2007 and no data of fructification were available after this date. In each forest plot, acorns were collected at the population scale, with ten 0.5 m^2^-litterfall traps set up under the closed canopy and evenly distributed over an area of about half a hectare. The litter fallen into the traps was collected each season, and sorted by distinguishing the leaves, branches and acorns from oak trees. Acorns were then separated from their cupule and oven-dried. The dry mass of acorns was measured, then divided by the total area of all the traps and expressed in kg/ha. In addition, the mean diameter of trees in each population were assessed every five years from 1991 to 2014. We then estimated it every year using linear regressions. Daily mean, minimal and maximal temperatures (°C) and precipitation (mm) were extracted from the SAFRAN^52^ spatially explicit database (8 × 8 km grid) for each site.

The elevation gradient survey was set up in the French Pyrenees, along a replicated transect in two parallel valleys: Ossau and Gaves (latitude 42°47′N to 43°45′N; longitude 00°44′W to 00°06′E). Five natural mature populations of *Q*. *petraea* were monitored in each valley, at different elevations, from 131 m to 1630 m (Table S5). At each site, nets were set up 1 m above the ground under the whole tree canopy, to collect all the acorns produced by an individual. In total 15, 13, 25 and 30 adult trees were monitored in 2012, 2013, 2014 and 2015, respectively. The 30 trees had a mean height of 19.2 ± 9.4 m and a mean diameter of 37.7 ± 19.6 cm. From 2012 onwards, the organic components (leaves, branches, fruits) fallen from the trees were harvested every two weeks, from the end of September until the beginning of December. For each tree, the projected area of the canopy on the ground (SC_OBi_) was calculated by first defining the canopy center (O) and then determining the distance from O to the outer limit of the canopy (B_i_), at 8 points, 45° apart (OB_1–8_). The surface area was calculated as:

1 $$S{C}_{O{B}_{i}}=\frac{\pi }{i}\times \sum O{B}_{i}^{2}$$

The harvested litters were sorted in the laboratory, and total acorn production, total dry mass and mean acorn weight per tree and per year (g) were determined. Total seed production was normalized by dividing by the total projected surface area of the tree canopy. Air temperature was measured with a data logger (HOBO Pro RH/Temp, Onset Computer Corporation, Bourne, Massachusetts, USA) at all sites. Data were recorded hourly, from January 1 2012 to December 31 2015.

### Statistical analysis

#### Temporal trend

We evaluated the change in seed production over time separately for the two species, with a linear mixed-effects model:

2 $${Y}_{j\Theta }={a}_{\mu }+{b}_{\mu }\Theta +({a}_{j}+{b}_{j}\Theta )+{\varepsilon }_{j\Theta }$$

where *a*_*μ*_ and *b*_*μ*_ are respectively the overall intercept and the overall regression slope of acorn production regressed over time (*Θ*) *a*_*j*_ and *b*_*j*_ are the random population-specific intercept and slope deviations associated with population *j*,, and *ε*_*j*Θ_ are the residuals. For both species, we compared the model [2] with a simpler model not accounting for population random-deviation in slope *b*_*j*_. The fit of the two models was compared with a likelihood-ratio test. No significant variation in slope were detected for both species (*Q*. *petraea*: χ^2^ = 0.18, *p* = 0.91; *Q*. *robur*: χ^2^ = 0.00, *p* = 1) indicating that the temporal trends were similar among populations (the variance of *b*_*j*_ did not differ from zero). Parameters were thus estimated from the simpler model: ${Y}_{j\Theta }={a}_{\mu }+{b}_{\mu }\Theta +({a}_{j})+{\varepsilon }_{j\Theta }$.

In addition, we assessed change in seed production over time separately for the two species and compared linear mixed-effects models adding tree age and diameter as fixed effect to the simpler model [2]:

3 $${Y}_{j\Theta AD}={a}_{\mu }+{b}_{\mu }\Theta +{d}_{\mu }A+{g}_{\mu }D+({a}_{j})+{\varepsilon }_{j\Theta AD}$$

where *d*_*μ*_ and *g*_*μ*_ are the regression slopes of acorn production regressed respectively over the mean age of the population in 1994 (*A*) and over the mean diameter of the population estimated for every year (*D*). To determine which of the two covariates helps to explain better the variation in seed production, we compared models with and without the effects of tree age (*A*) and diameter (*D*) using the Akaike Information Criterion corrected for small sample size (AICc) (Table S1). Models with the lowest AICc were selected, however, we consider models with ΔAICc between 0 and 2 to have equivalent support^53^ and based on the principle of parsimony we selected the simplest one.

For *Q*. *petraea*, AICc differences gave support to the model with the fixed effect *d*_*μ*_ *A*, (Table S1), thus, we estimated the effect of age on acorn production (Table S2).

We then evaluated the response of seed production to temperature:

4 $${Y}_{ijT}={a}_{\mu }+{c}_{\mu }T+({a}_{j}+{c}_{j}T)+{\varepsilon }_{ijT}$$

where *c*_*μ*_ is the general regression slope of acorn production regressed against temperature (*T*), *a*_*j*_ is the random deviation associated with population *j*, and *c*_*j*_ is the random population-specific deviation in slope, and *ε*_*ijT*_ denotes the residuals. As before, we compared the model [4] with a simpler model not accounting for population random-deviation in slope (*c*_*j*_). No significant variation in slope were detected for both species (Likelihood-ratio tests: *Q*. *petraea*: χ^2^ = 2, *p* = 0.08; *Q*. *robur*: χ^2^ = 2, *p* = 0.12), indicating that the effect of temperature on acorn production was consistent among populations (Figure S5).

We therefore used the simplified model [4] (i.e. ${Y}_{ijtT}={a}_{\mu }+{c}_{\mu }T+({a}_{j})+{\varepsilon }_{ijT}$) to evaluate the effect of temperature on seed production. Model [4] was compared to a model without the temperature fixed effect *c*_*μ*_, using the Akaike Information Criterion corrected for small sample size (AICc). To determine the temperature variable having the strongest influence on seed production, this comparison was performed taking several estimations of annual temperature *T:* the mean temperature computed for each month and the mean temperature computed for every two month over the year. AICc differences giving support to the more complex model were much higher for the periods of April (AICc = 28.1), March-April (AICc = 17.2), and April-May (AICc = 18.8), for *Q*. *petraea* and for April (AICc = 4.9), and April-May (AICc = 5.2) for *Q*. *robur* (Figure S6). As the ΔAICc were higher for temperatures recorded during the spring months, we refined the analysis to different spring periods (Table S7). For *Q*. *petraea*, a significant regression slope was found with the temperature in April (*c*_*μ*_ = 94.8 [61.5, 128.4] kg/ha/°C), March-April (*c*_*μ*_ = 103.0 [56.6, 152.4] kg/ha/°C), April-May (*c*_*μ*_ = 111.9 [63.1, 146.1] kg/ha/°C) and May-June (*c*_*μ*_ = 41.6 [1.1, 86.1] kg/ha/°C). For *Q*. *robur*, a significant regression slope was observed only for April (*c*_*μ*_ = 54.07 [14.4, 93.7] kg/ha/°C) and April-May (*c*_*μ*_ = 72.66 [19.6, 120.5] kg/ha/°C (Tables 1 and S7). The mean temperatures during the periods of April and April-May are determinant for seed production in both species. We represented April-May period in Fig. 2 as it covered a larger period.

To evaluate the effect of precipitation on fructification, we compared model [4] with a similar mixed effect model taking into account the sum of precipitations as a fixed effect in addition to the spring temperature. The inclusion of the five precipitation periods: winter, (January to March), spring (April to June), summer (July to September), autumn (October to December) and the whole year, as predictor were compared with model [4] using AICc.

As spring temperature was found to have increased over the last decades (Figure S4) and seed production was significantly correlated with temperature (Table 1 and Fig. 2), we considered the observed temporal trend in seed production in both species (Table 1 and Fig. 1) to be due principally to the increase in temperature. However, as the populations were distributed over a large area covering a large range of temperatures, we explicitly accounted for variability due to the year and population, with the following multiple regression model:

5 $${Y}_{ij{T}_{P}{T}_{Y}}={a}_{\mu }+{b}_{\mu }{T}_{P}+{c}_{\mu }{T}_{Y}+({a}_{j})+{\varepsilon }_{ij{T}_{P}{T}_{Y}}$$

where *b*_*μ*_ denotes the overall slope of the mean population temperature regressed against year (*T*_*P*_) and *c*_*μ*_ is the overall slope of mean yearly temperature regressed against population (*T*_*Y*_) for acorn production. *a*_*j*_ is the population random intercept and and ${\varepsilon }_{ij{T}_{P}{T}_{Y}}$ denotes the residuals. We used model [5], with the mean temperature of April-May (Table S4).

#### Trend along the elevation gradient

We then evaluated the sensitivity of reproduction to temperature along the elevation gradients in Pyrenees. We used the following mixed-effect model:

6 $${Y}_{ijkT}={a}_{\mu }+{c}_{\mu }T+({p}_{j})+({n}_{k(j)})+{\varepsilon }_{ijkT}$$

where *a*_*μ*_ denotes the overall intercept and *c*_*μ*_ the overall regression slope of acorn production against temperature (*T*), *p*_*j*_ and *n*_*k*(*j*)_ are the random deviations associated with population *j* and the individuals k within population *j*, respectively, and the residuals are denoted *ε*_*ijKT*_. We tested the effect of temperature over the same period as above (Tables 1 and S7).

Finally, using the same model [6], we also evaluated the sensitivity of acorn size to temperature along the same elevation gradient.

All the linear mixed effects models were fitted by the restricted maximum likelihood (REML)^54^ method in the *lme4* R package^55^.

## Electronic supplementary material

supplementary information
