# Supplementary material for: Increasing spring temperatures favor oak seed production in temperate areas
Source: Sci Rep. 2017 Aug 17;7:8555. doi: 10.1038/s41598-017-09172-7 (PMC5561138; doi:10.1038/s41598-017-09172-7)
Supplement: Supplementary file 1 — supplementary information [file 41598_2017_9172_MOESM1_ESM.pdf]

SUPPLEMENTARY INFORMATIONS

Increasing spring temperatures favor oak seed production in temperate areas

Authors:

Thomas Caignard[1], Antoine Kremer[1], Cyril Firmat[1,2], Manuel Nicolas[3], Samuel Venner[4], Sylvain Delzon[1]

Affiliations:

1- BIOGECO, INRA, Univ. Bordeaux, 33615 Pessac, France

2- INRA, URP3F, RD150. Site du Chêne, BP 86006, 86600 Lusignan, France.

3- Office National des Forêts, Département recherche-développement-innovation, Boulevard de Constance, 77300 Fontainebleau, France.

4- Laboratoire de Biométrie et Biologie Evolutive UMR 5558-CNRS, Université de Lyon, Université Claude Bernard Lyon 1, Villeurbanne F-69365 Lyon, France.

Table S1 | Model selection for testing the effect of age and diameter on acorn production in both species: *Quercus petraea* and *Quercus robur*. AICc values were obtained from model fitted for maximum likelihood (ML). For each model the random factor was the population. Models are ranked by AICc values.

Species	Linear mixed effect model	K	AICc	ΔAICc	AICc weight
Quercus petraea	Year + Age + Diameter	6	3880.23	0	0.53
	Year + Age	5	3881.29	1.06	0.31
	Year	4	3883.34	3.11	0.11
	Year + Diameter	5	3885.33	5.1	0.04
	Constant	3	3892.49	12.26	0
Quercus robur	Year	4	1850.03	0	0.31
	Year + Diameter	5	1850.42	0.39	0.26
	Constant	3	1850.98	0.95	0.19
	Year + Age	5	1851.5	1.47	0.15
	Year + Age + Diameter	6	1852.48	2.45	0.09

Table S2 | Slopes of the linear mixed-effect regression between acorn production in kilograms per hectare and year ($b_\mu \Theta$) and the mean age of the population in 1994 ($d_\mu A$) for *Q. petraea*. Bold characters correspond to significant regression slopes. The 95% confidence intervals are indicated in square brackets.

	Quercus petraea
b_μ	**19.73 [8.29, 31.16]**
d_μ	**-1.89 [-3.71, -0.07]**

Table S3 | Comparison of Model [4] with a similar mixed effect model taking into account, in addition to spring temperature, the sum of precipitation as a fixed effect for different seasons and for both species (a and b). Comparison were carried out using the Akaike Information Criterion corrected for small sample size (AICc).

Species	Period of the year	Linear mixed effect model	K	AICc	ΔAICc	AICc weight
Quercus petraea	Winter	Temperature	4	3873.68	0	0.65
		Temperature + Precipitation	5	3874.9	1.22	0.35
	Spring	Temperature	4	3873.68	0	0.66
		Temperature + Precipitation	5	3875.04	1.36	0.34
	Summer	Temperature	4	3873.68	0	0.54
		Temperature + Precipitation	5	3873.97	0.28	0.46
	Autumn	Temperature	4	3873.68	0	0.58
		Temperature + Precipitation	5	3874.3	0.62	0.42
	Year	Temperature	4	3873.68	0	0.74
		Temperature + Precipitation	5	3875.75	2.06	0.26
Quercus robur	Winter	Temperature	4	1845.82	0	0.71
		Temperature + Precipitation	5	1847.61	1.8	0.29
	Spring	Temperature	4	1845.82	0	0.7
		Temperature + Precipitation	5	1847.48	1.66	0.3
	Summer	Temperature	4	1845.82	0	0.71
		Temperature + Precipitation	5	1847.62	1.8	0.29
	Autumn	Temperature	4	1845.82	0	0.73
		Temperature + Precipitation	5	1847.81	2	0.27
	Year	Temperature	4	1845.82	0	0.75
		Temperature + Precipitation	5	1847.99	2.17	0.25

Table S4 | Regression slopes of the linear mixed-effect regression between acorn production in kilograms per hectare and b_μ the mean population temperature regressed against year (T_P) and c_μ the mean yearly temperature regressed against population (T_Y) estimated from model [4] for each species. Bold characters correspond to significant regression slopes. The 95% confidence intervals are indicated in square brackets.

	Quercus petraea	*Quercus robur*
b_μ	10.53 [-68.0, 89.0]	5.17 [-63.1, 73.4]
c_μ	**147.78 [90.7, 205.0]**	**119.78 [36.2, 203.3]**

Table S5 | Site description and climate for the temporal gradient

Species	Station	Latitude	Longitude	Altitude (m)	Temperature (°C)	Precipitation (mm)
Quercus petraea	CHS 81	44°02‘N	01°44‘E	300	12.28	848.1
Quercus petraea	CHS 01	46°10‘N	05°14‘E	260	11.04	992.2
Quercus petraea	CHS 86	46°37‘N	00°29‘E	116	12.13	789.3
Quercus petraea	CHS 03	46°40‘N	02°43‘E	260	11.53	776
Quercus petraea	CHS 58	46°58‘N	03°39‘E	270	11.41	943.7
Quercus petraea	CHS 21	47°04‘N	05°04‘E	220	11.04	780.3
Quercus petraea	CHS 18	47°15‘N	02°07‘E	176	11.39	839.3
Quercus petraea	CHS 41	47°34‘N	01°15‘E	127	11.58	656.3
Quercus petraea	CHS 68	47°41‘N	07°28‘E	256	10.43	845.2
Quercus petraea	CHS 72	47°47‘N	00°22‘E	170	11.64	766.5
Quercus petraea	CHS 88	48°01'‘N	06°02‘E	330	9.97	1068.5
Quercus petraea	CHS 35	48°10‘N	01°32‘W	80	11.47	807.1
Quercus petraea	CHS 10	48°17‘N	04°27‘E	160	11.04	792.4
Quercus petraea	CHS 61	48°31‘N	00°40‘E	220	10.74	808.5
Quercus petraea	CHS 57a	48°52‘N	06°29‘E	315	10.36	863.3
Quercus petraea	CHS 57b	49°00‘N	07°27‘E	320	9.85	863
Quercus petraea	CHS 51	49°02‘N	04°57‘E	180	10.52	864.7
Quercus petraea	CHS 27	49°21’N	01°30’E	175	10.29	794.6
Quercus petraea	CHS 60	49°23‘N	02°18‘E	55	10.81	695
Quercus robur	CHP 65	43°12'N	00°02‘W	370	11.9	997.5
Quercus robur	CHP 40	43°44'N	00°50'W	20	13.52	987.7
Quercus robur	CHP 18	46°49'N	02°34‘E	175	11.5	827
Quercus robur	CHP 71	46°58'N	05°14‘E	190	11.2	773.9
Quercus robur	CHP 49	47°27'N	00°01‘EW	57	12.23	704.9
Quercus robur	CHP 70	47°52'N	06°12‘E	240	10.44	1167.7
Quercus robur	CHP 10	48°20'N	04°18‘E	115	11.04	710.9
Quercus robur	CHP 55	49°01'N	05°46‘E	220	10.12	844.6
Quercus robur	CHP 59	50°10'N	03°45'E	149	10.15	767.3

Table S6 | Site description and climate for the spatial gradient

Valley	Station	Species	Latitude	Longitude	Altitude (m)	Gradient (m)	Mean annual temperatures (°C)			
							2012	2013	2014	2015
Luz	Laveyron	*Quercus petraea*	43°45’N	00°13’E	131	100	13.5	12.8	13.7	13.5
Luz	Ibos	*Quercus petraea*	43°15’N	00°00’W	387	400	12.5	12.11	12.8	13.3
Luz	Chèze	*Quercus petraea*	42°55’N	00°02’W	803	800	10.2	10.4	11.5	11.9
Luz	Gèdre Bas	*Quercus petraea*	42°47’N	00°25’W	1235	1200	9.2	8.7	9.8	10.7
Luz	Péguère	*Quercus petraea*	42°52’N	00°07’W	1630	1600	7.8	6.9	8.1	8.8
Ossau	Josbaig	*Quercus petraea*	43°13’N	00°44’W	259	100	11.7	12.9	14	13.7
Ossau	Bager	*Quercus petraea*	43°07’N	00°32’W	422	400	10.8	11.7	12.7	12.5
Ossau	Le Hourcq	*Quercus petraea*	42°54’N	00°26’W	841	800	9.4	8.4	9.7	9.7
Ossau	Gabas	*Quercus petraea*	42°53’N	00°25’W	1194	1200	8.9	8.3	9.5	9.9
Ossau	Artouste	*Quercus petraea*	42°53’N	00°24’W	1614	1600	7.1	6.2	7.7	8.3

Table S7 | Regression slopes of the linear mixed-effect regression between acorn production (kg/ha) and mean temperature (°C) for different periods of time within the spring season. Regression slopes were estimated from model [3] for the temporal gradient and model [5] for the spatial gradient. Regression slopes and their confidence intervals, and R^2 values are indicated. Bold characters correspond to significant regression slopes.

Species	Period	Temporal		Spatial	
		Slope	R^2	Slope	R^2
Quercus petraea	March	16.97 [-13.9, 47.8]	0.04	12.17 [-4.9, 25.7]	0.04
	April	**94.88 [61.5, 128.4]**	0.64	**22.25 [10.0, 35.6]**	0.16
	May	29.97 [-9.1, 69.0]	0.09	**24.12 [12.9, 36.7]**	0.25
	June	24.13 [-3.8, 52.2]	0.20	**29.13 [12.8, 49.4]**	0.24
	March - April	**102.98 [56.6, 152.4]**	0.41	**20.38 [6.3, 35.4]**	0.10
	April - May	**111.89 [63.1, 146.1]**	0.73	**33.42 [17.6, 58.9]**	0.33
	May - June	**41.59 [1.1, 86.1]**	0.30	**28.16 [14.4, 44.7]**	0.26
Quercus robur	March	-1.72 [-36.0, 32.5]	0	-	-
	April	**54.07 [14.4, 93.7]**	0.45	-	-
	May	45.36 [-2.2, 93.0]	0.25	-	-
	June	5.88 [-31.1, 42.8]	0	-	-
	March - April	32.58 [-13.3, 78.5]	0.08	-	-
	April - May	**72.66 [19.6, 120.5]**	0.34	-	-
	May - June	31.03 [-19.5, 81.6]	0.08	-	-

Figure S1 | Localization of the *Quercus petraea* (orange) and *Quercus robur* (blue) populations of the temporal gradient. The base map featuring the forested areas was extracted free of charge and use from the IGN National Forest Inventory cartography site (IGN 2015 – Départements administratifs, GEOFLA® 2.1). The map was generated using the free and open source geographic information system Qgis 2.6[56].

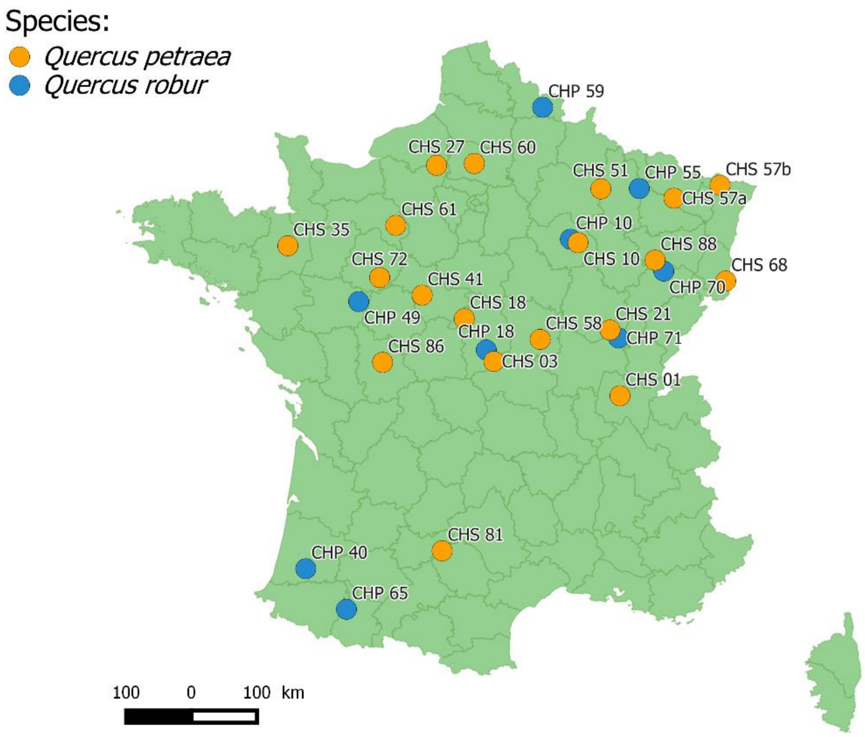

Figure S2| Patterns of acorn production from 1994 to 2007 for each population of the temporal gradient (RENECOFOR). Each dot corresponds to the amount of acorn produced in kilogram per hectare for each site per year.

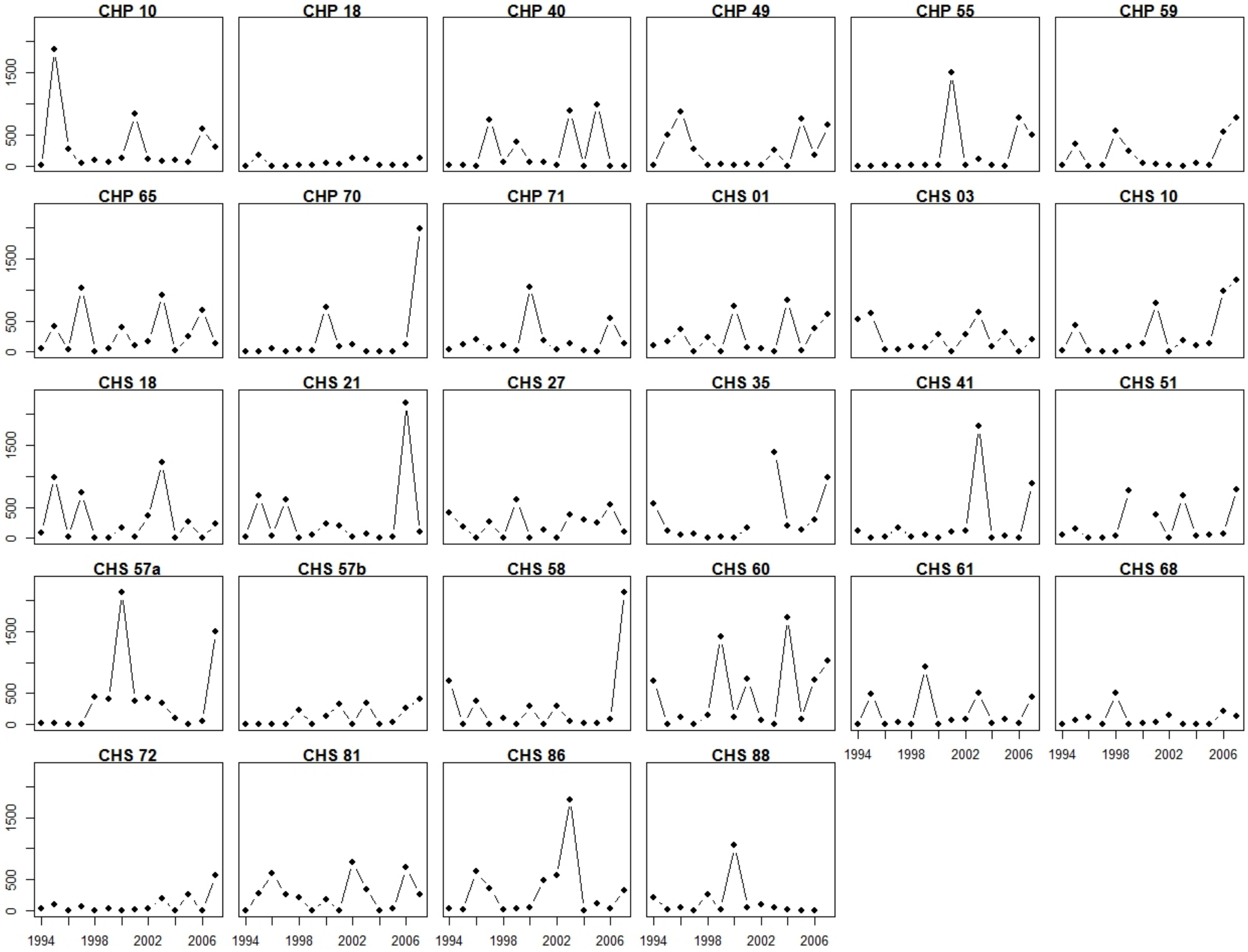

Figure S3 | Changes in the degree of reproductive synchrony, measured by the coefficient of Spearman, according to the distance between populations. The degree of synchrony is plotted for *Quercus petraea* (a) and *Quercus robur* (b). The slope of the regression line, calculated from a linear model and represented for every 100 km, are given for both species with the coefficient of determination (R^2).

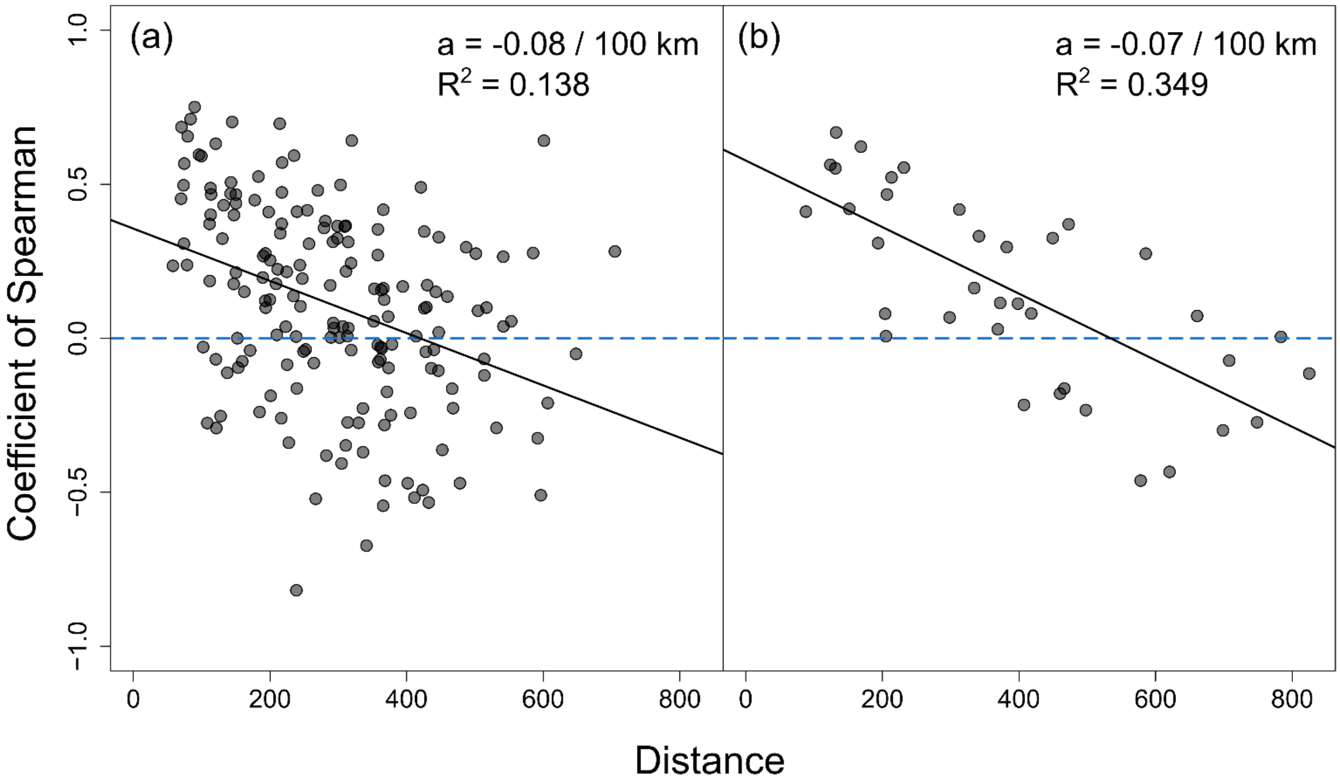

Figure S4 | We evaluated the change in temperature from 1962 to 2012 separately for the two species populations with a linear mixed effect model:

$$Z_{jt} = a_{\mu} + b_{\mu}t + (a_j) + \varepsilon_{jt}$$

with a_{μ} denoting the overall intercept and b_{μ} the overall slope of time (t) on temperature, a_j the random deviation associated to population j, and the residuals ε_{jt} Slopes and confidence intervals are estimated from the previous model and are represented in the following figure. Each dot represents the average temperature of each sites for one year on both species. The coefficient of determination (R^2) calculated between the previous model and the mean temperature of each site are indicated. Years of seed production monitoring are indicating between the two vertical dashed lines.

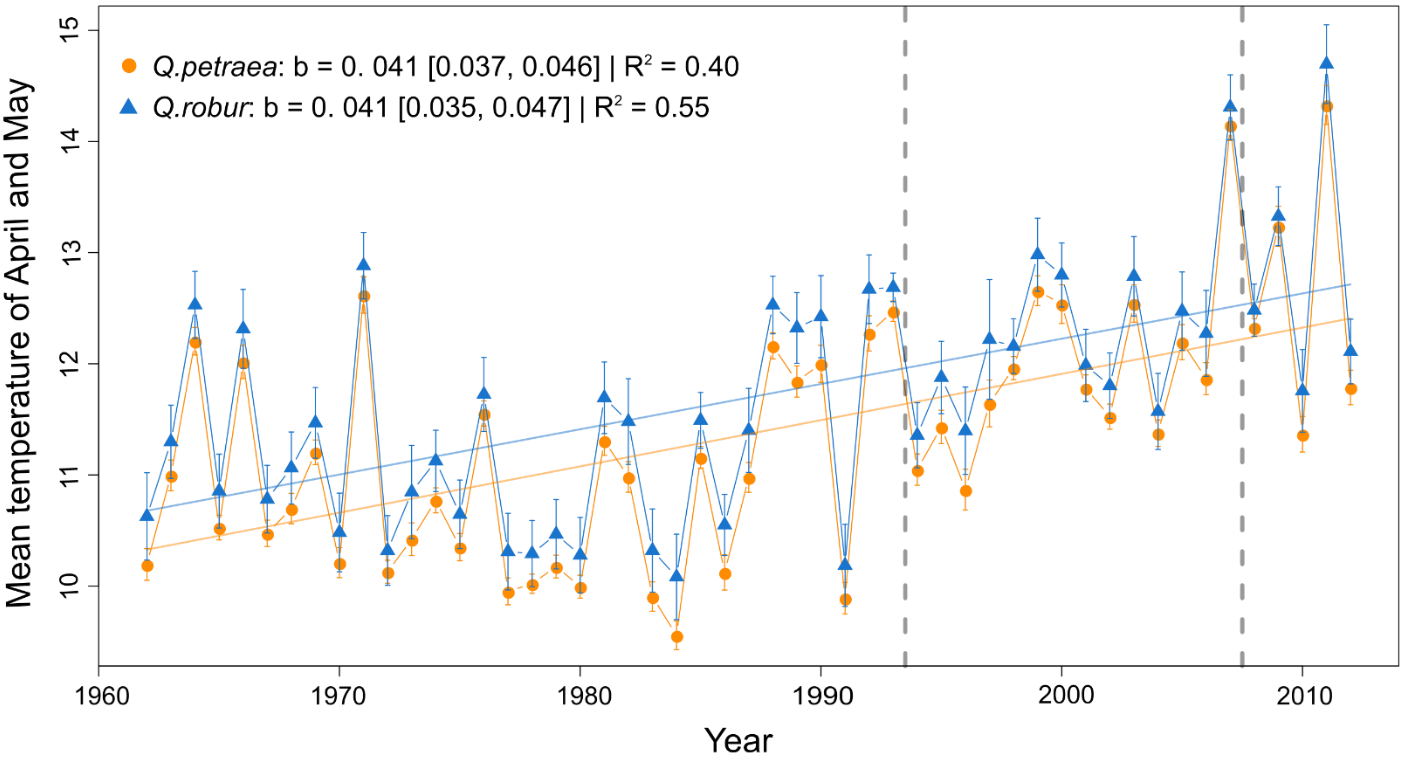

Figure S5 | Relationships between acorn production and mean spring temperature for each population of *Quercus petraea* (a) and *Quercus robur* (b). For both species, acorn production data for each population and year were binned into 19 temperature classes of approximately the same size, 14 for *Q.petraea* and 6 or 7 for *Q.robur*. The temporal trends in acorn production for each population are indicated by each grey lines.

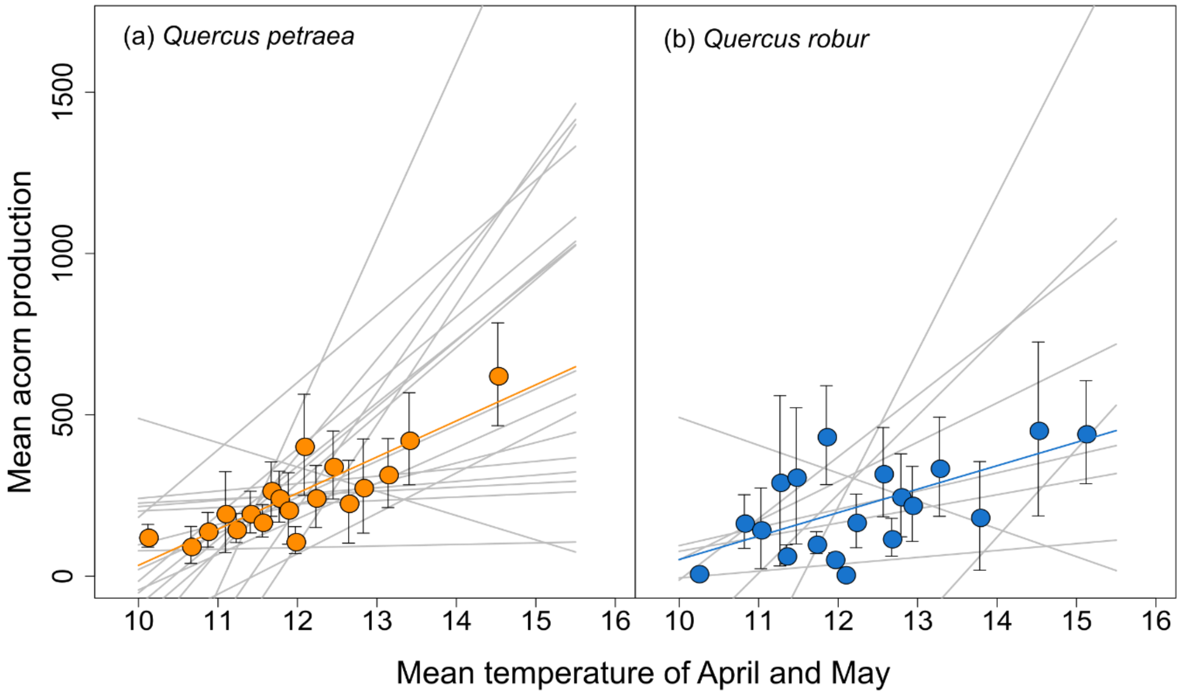

Figure S6| Comparison of Model [3] with the Null Model (i.e. without the fixed temperature effect implemented) using the Akaike Information Criterion corrected for small sample size (AICc), tested for each month and every two months on *Q. petraea* (a and c) and *Q. robur* (b and d). The dashed red line represents an AICc of 4, considered as an empirical level for which support to the null model is considerably low[53].

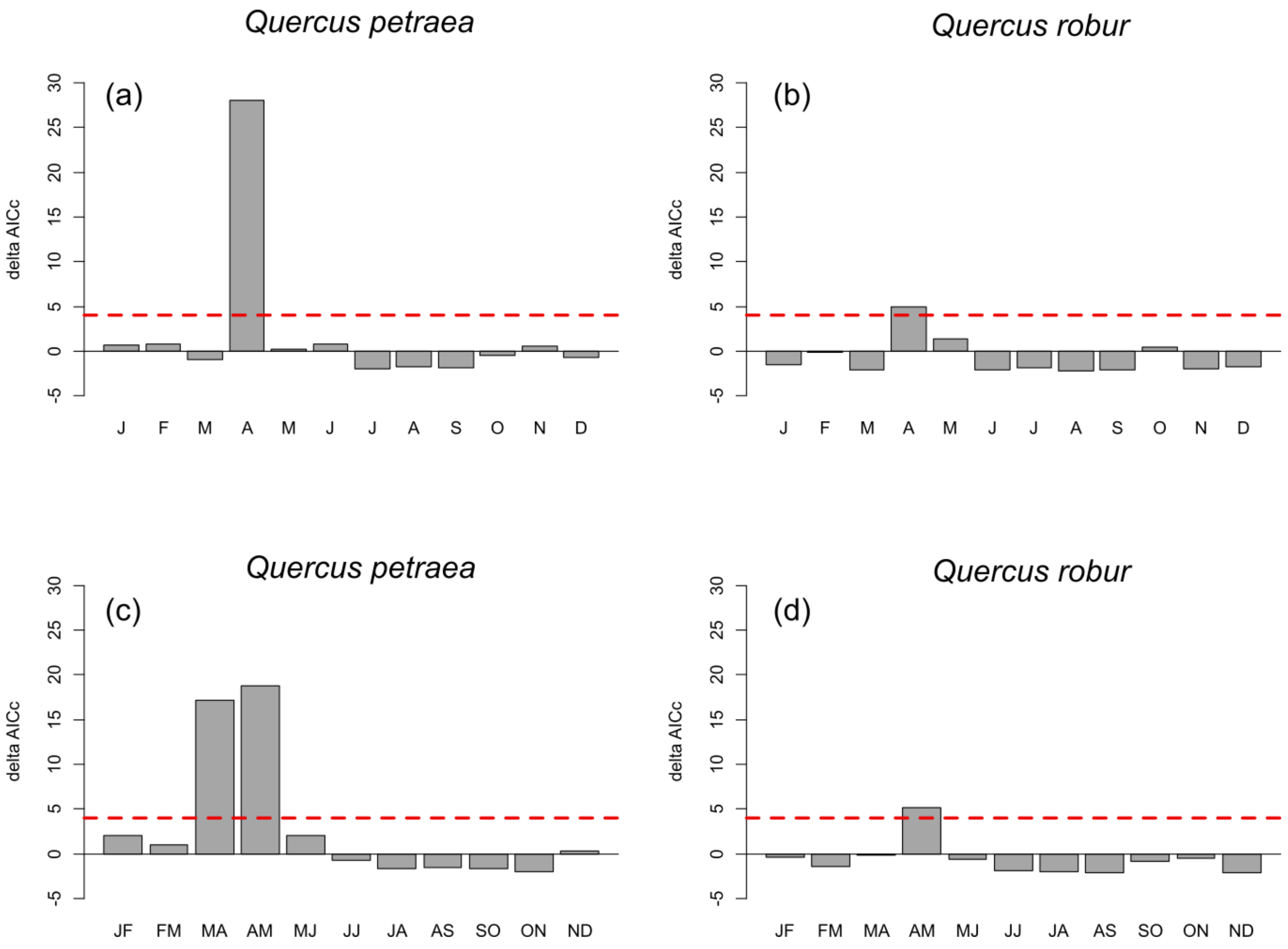

References

53. Burnham, K.P., and Anderson, D.R. Model Selection and Multimodel Inference: A Practical Information-Theoretic Approach (Springer Science & Business Media) (2003).

56. Quantum GIS Development Team. Quantum GIS Geographic Information System. Open Source Geospatial Foundation Project. http://qgis.osgeo.org (2015).
